# Supplementary figures and images for: Cortical stiffness of keratinocytes measured by lateral indentation with optical tweezers
Source: PLoS One. 2020 Dec 31;15(12):e0231606. doi: 10.1371/journal.pone.0231606 (PMC7774922; doi:10.1371/journal.pone.0231606)

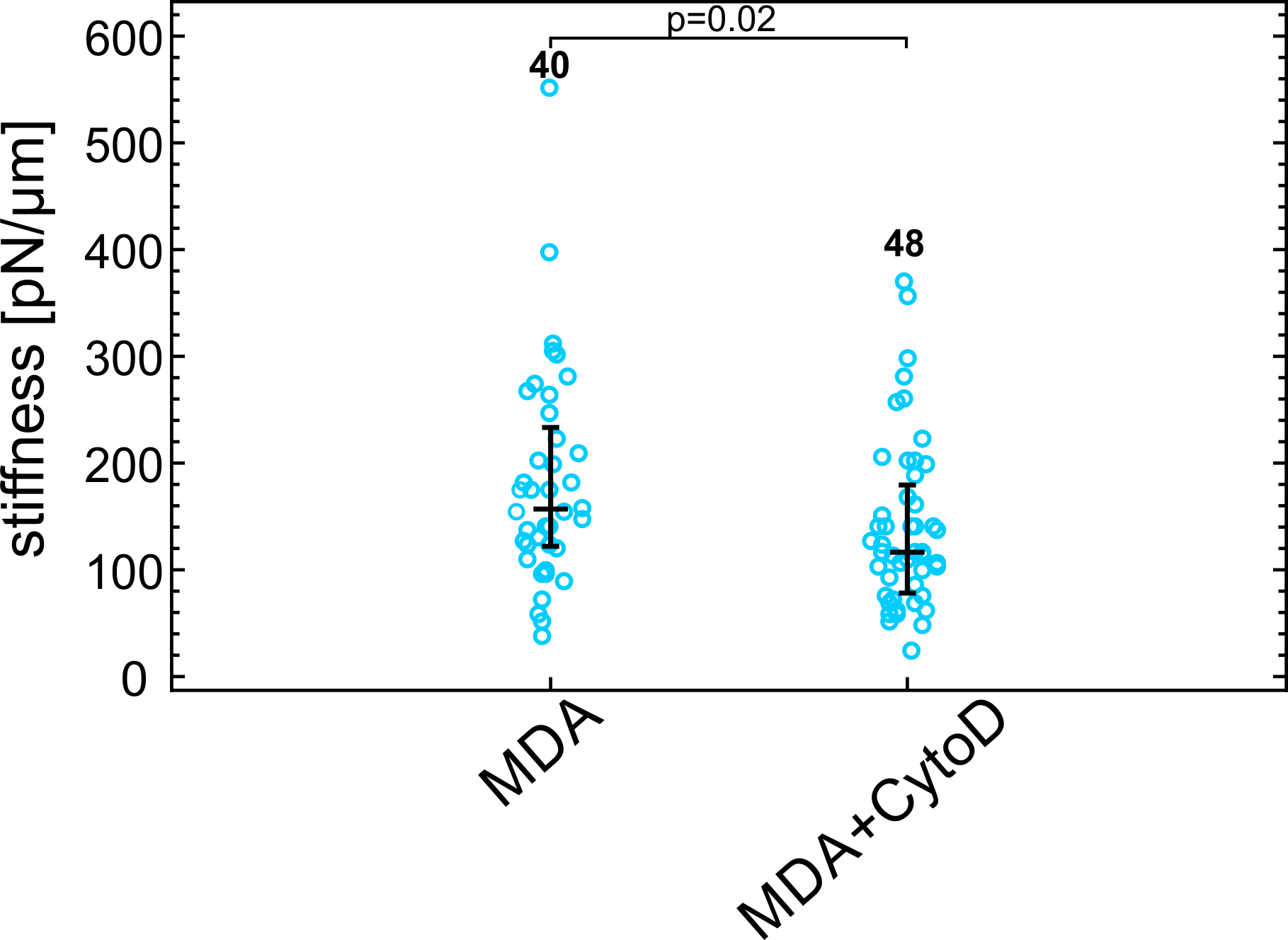

Supplement: S1 Fig — The relative stiffness of breast cancer MDA cells before and after the treatment with Cytochalasin D, which disrupts the actin skeleton and softens the cells. After the treatment, the median stiffness decreased for 25% from 156 pN/μm to 117 pN/μm (p = 0.02). The stiffness was measured at the deformation rate of 1 μm/s. The median value and the quartiles are indicated and the number of measured cells is denoted above the data points. (TIF) [file pone.0231606.s001.tif]

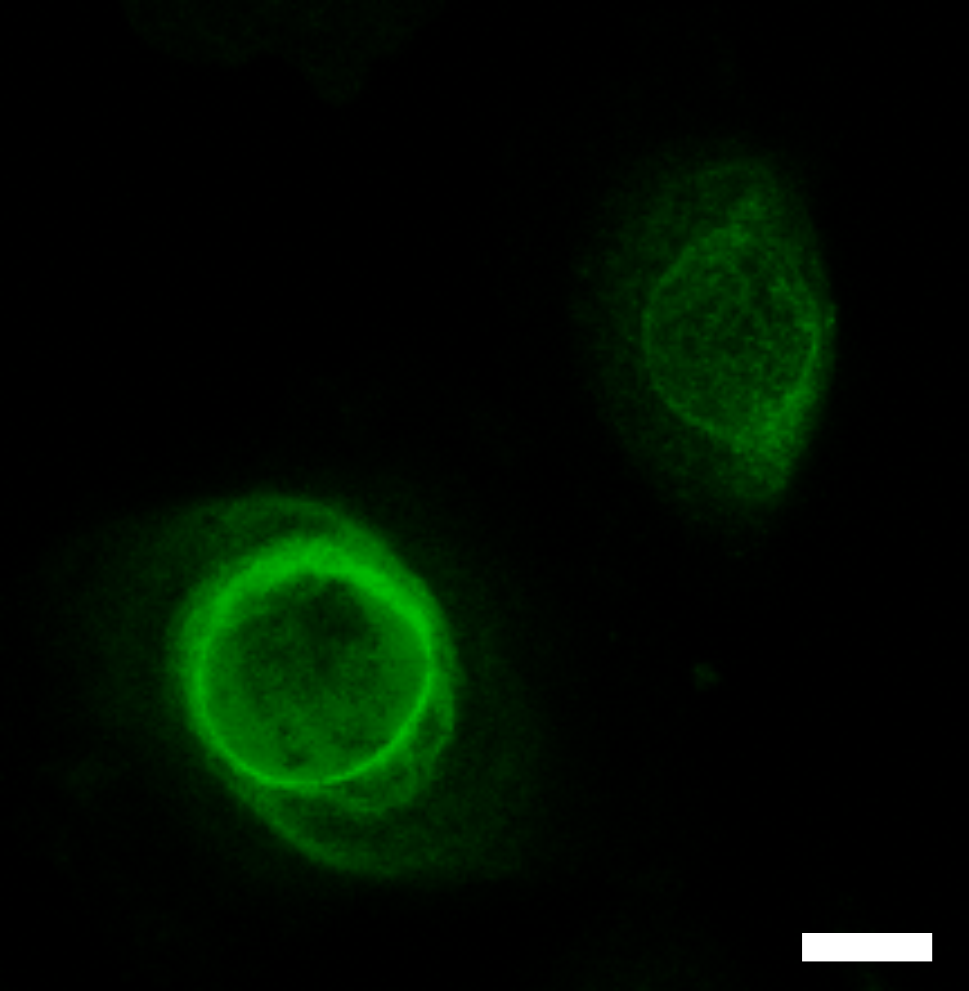

Supplement: S2 Fig — The scale bar represents 10 μm. (TIF) [file pone.0231606.s002.tif]

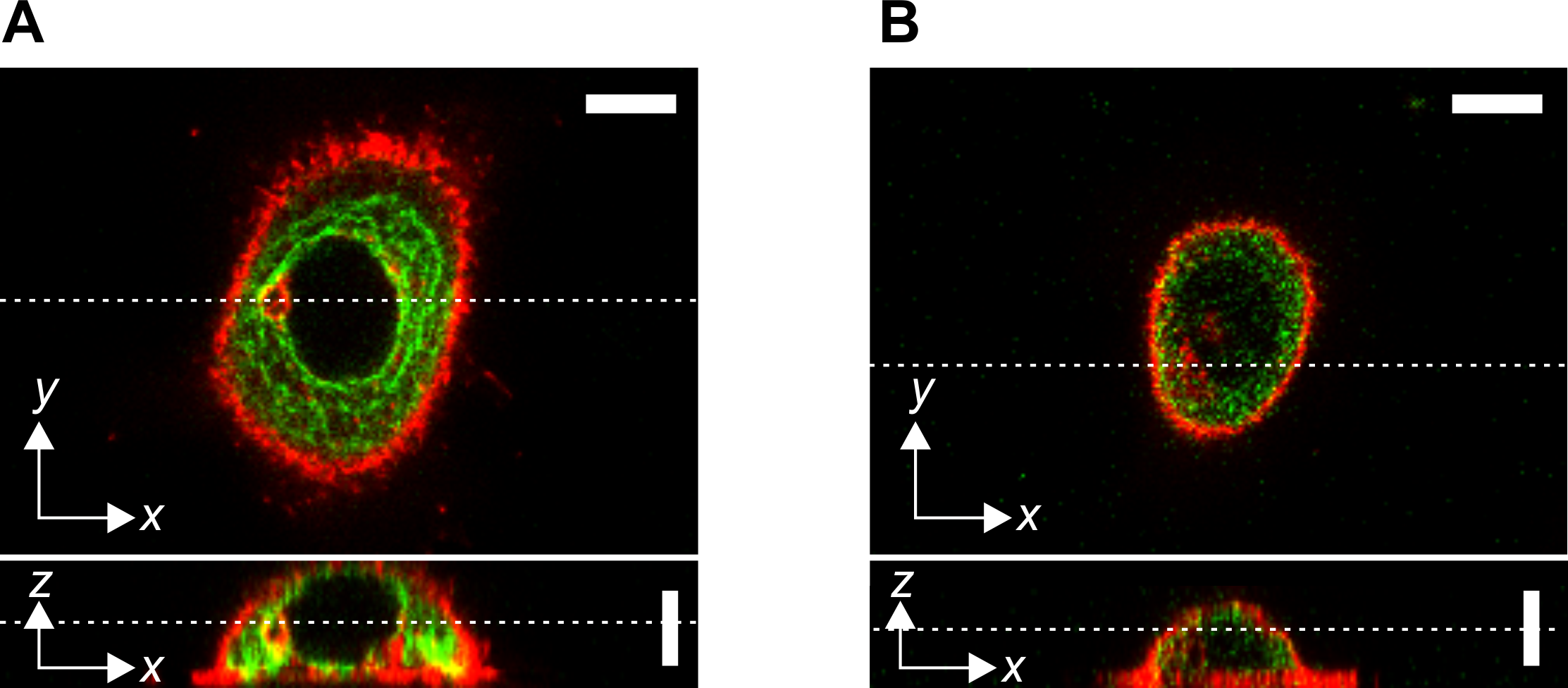

Supplement: S3 Fig — (A) A typical NEB1 cell and (B) a typical KEB7 cell. In both cell types, the keratin signal is located in the cell interior underneath the cortex and there is no visible colocalization of the keratin and actin signals. The white dashed lines represent the section planes at the point of indentation experiments and scale bars represent 10 μm. (TIF) [file pone.0231606.s003.tif]

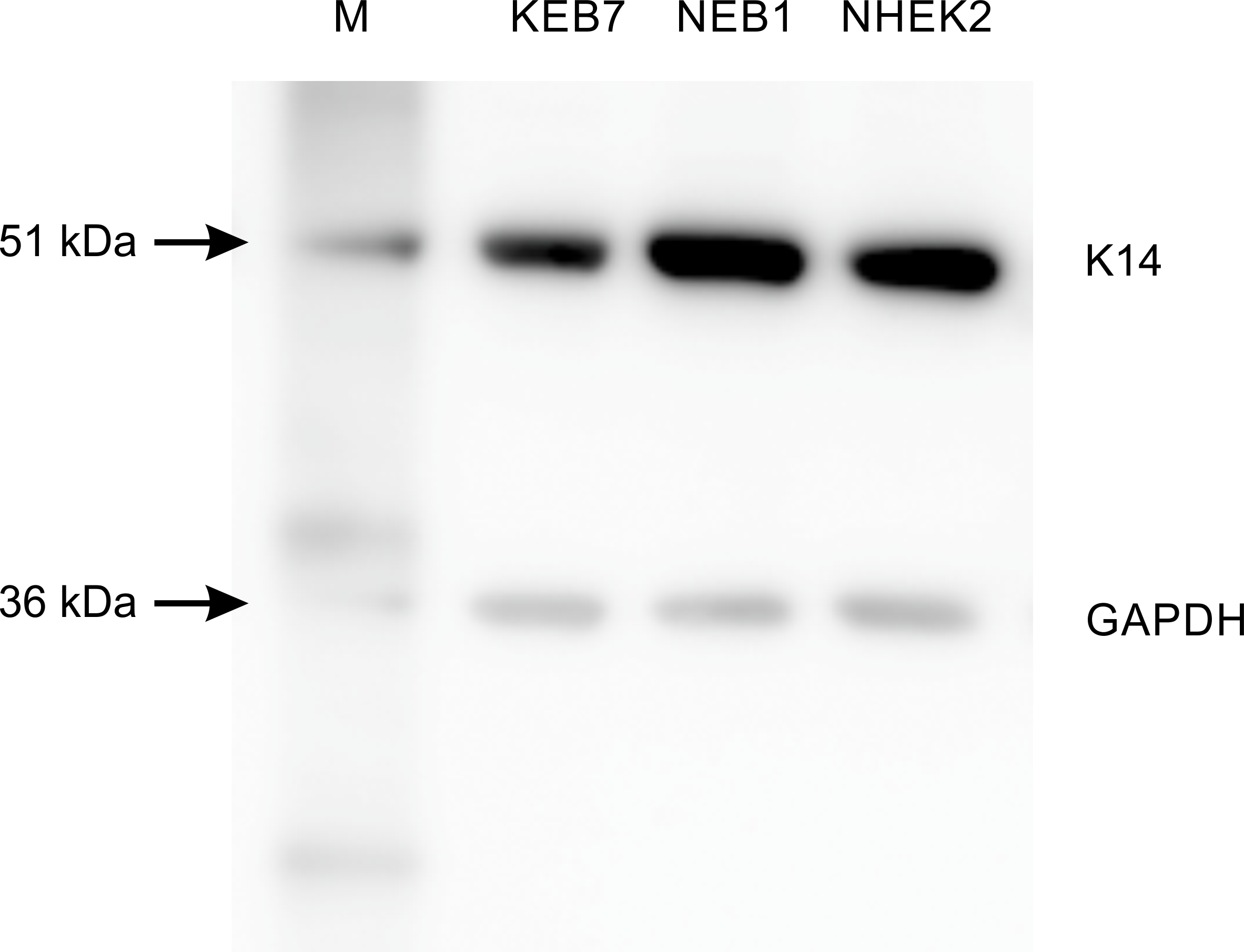

Supplement: S4 Fig — Keratin 14 protein expression in NEB1, KEB7 and NHEK2 keratinocytes were analyzed by Western blot. Quantification showed that mutant KEB7 cells exhibited approximately 5 times less keratin 14 than NEB1 cells and the latter had approximately 20% less keratin 14 than NHEK2 cells. M—MagicMark XP standard. (TIF) [file pone.0231606.s004.tif]

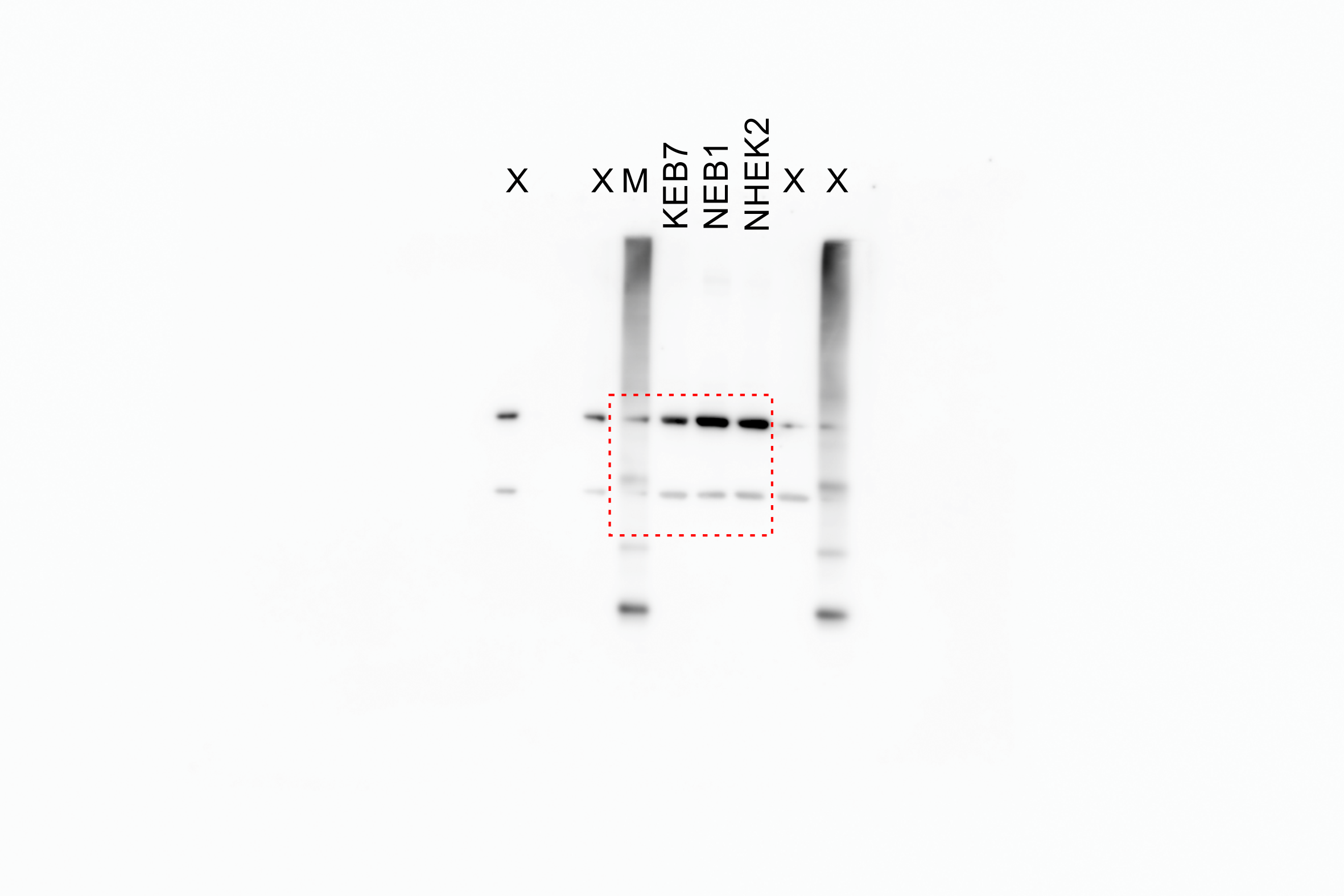

Supplement: S1 Raw image — (TIF) [file pone.0231606.s005.tif]
