# Supplementary material for: Cortical stiffness of keratinocytes measured by lateral indentation with optical tweezers
Source: PLoS One. 2020 Dec 31;15(12):e0231606. doi: 10.1371/journal.pone.0231606 (PMC7774922; doi:10.1371/journal.pone.0231606)
Supplement: S1 Table — (DOCX) [file pone.0231606.s006.docx]

**S1 Table. A summary of the results presented in Fig 4.**

| **Cell Type** | **NEB1** | | **KEB7** | | **NHEK2** | | **fixed NEB1** |
| --- | --- | --- | --- | --- | --- | --- | --- |
| deformation rate [µm/s] | 0.1 | 1 | 0.1 | 1 | 0.1 | 1 | 1 |
| N | 29 | 21 | 71 | 40 | 42 | 20 | 21 |
| median value [pN/µm] | 78 | 105 | 132 | 141 | 218 | 170 | 366 |
| first quartile [pN/µm] | 70 | 84 | 73 | 58 | 130 | 142 | 261 |
| third quartile [pN/µm] | 90 | 135 | 207 | 217 | 336 | 272 | 566 |
