# Supplementary material for: Cortical stiffness of keratinocytes measured by lateral indentation with optical tweezers
Source: PLoS One. 2020 Dec 31;15(12):e0231606. doi: 10.1371/journal.pone.0231606 (PMC7774922; doi:10.1371/journal.pone.0231606)
Supplement: Attachment — Submitted filename: Answers to the Reviewer_.docx [file pone.0231606.s011.docx]

We would like to thank the Reviewer #2 for his very thorough overview of our manuscript. Both mistakes he pointed out were the results of our inaccuracy.

- Lines 218-224: the values written in these lines are correct and represent the mean of three independent Western Blots we made. In the lines 544-547 we forgot to update the numbers, which we corrected now.
- Line 117: SDS-PAGE is a gradient gel (4-12%) and not a 20% SDS-PAGE, which we corrected now.
